# Curcumin Downregulates Human GM3 Synthase (hST3Gal V) Gene Expression with Autophagy Induction in Human Colon Carcinoma HCT116 Cells

**DOI:** 10.1155/2018/6746412

**Published:** 2018-11-11

**Authors:** Miri Lee, Hyunju Choi, Kyoung-Sook Kim, Dong-Hyun Kim, Cheorl-Ho Kim, Young-Choon Lee

**Affiliations:** ^1^Department of Medicinal Biotechnology, College of Health Sciences, Dong-A University, Busan 49315, Republic of Korea; ^2^Molecular and Cellular Glycobiology Unit, Department of Biological Sciences, Sungkyunkwan University, Kyunggi-Do 16419, Republic of Korea

## Abstract

Our recent report showed that curcumin, polyphenolic compound isolated from the herb* Curcuma longa*, upregulated the gene expression of human GD3 synthase (hST8Sia I) responsible for ganglioside GD3 synthesis with autophagy induction in human lung adenocarcinoma A549 cells. In this study, on the contrary to this finding, we demonstrated that curcumin downregulated the gene expression of human GM3 synthase (hST3Gal V) catalyzing ganglioside GM3 synthesis with autophagy induction in human colon carcinoma HCT116 cells. To clarify the mechanism leading to the downregulation of hST3Gal V gene expression in curcumin-treated HCT116 cells, we analyzed the curcumin-inducible promoter of the hST3Gal V gene by luciferase reporter assays. Promoter deletion analysis demonstrated that the -177 to -83 region, which includes putative binding sites for transcription factors NFY, CREB/ATF, SP1, EGR3, and MZF1, acts as the curcumin-responsive promoter of the hST3Gal V gene. Site-directed mutagenesis and chromatin immunoprecipitation analysis demonstrated that the CREB/ATF binding site at -143 is pivotal for curcumin-induced downregulation of hST3Gal V gene in HCT116 cells. The transcriptional activation of hST3Gal V in HCT116 cells was significantly repressed by an inhibitor of AMP-activated protein kinase (AMPK). These results suggest that AMPK signal pathway mediates hST3Gal V gene expression in HCT116 cells.

## 1. Introduction

Gangliosides, the sialylated glycosphingolipids, are components of lipid rafts along with sphingomyelin and cholesterol as well as a part of the glycocalyx in mammalian cells [1, 2]. Their biosynthesis occurs through stepwise addition of carbohydrate residues from their nucleotide sugar donors to the growing glycan by a series of specific glycosyltransferases in the Golgi apparatus [3, 4]. They are expressed in a cell type-specific and stage-specific manner and play crucial roles in numerous cellular events, including cell-cell interaction, cell differentiation, adhesion, signal transduction, and oncogenesis [4–7].

Recently, it was reported that gangliosides played an important role in autophagy induction [8–10]. For example, treatment with gangliosides mixture or ganglioside GT1b induced the reactive oxygen species (ROS)-mediated autophagic cell death via AKT-mTOR and ERK pathways in astrocytes [8]. In addition, these gangliosides have shown to induce autophagic cell death via the activation of IKK/NF-*κ*B pathway in astrocytes [9]. It was demonstrated that ganglioside GD3 is involved in autophagosome formation and maturation in human primary fibroblasts [10]. Although the roles of exogenous ganglioside in autophagy induction are known, no studies have been reported on the molecular mechanisms responsible for gene expression of ganglioside synthases catalyzing ganglioside biosynthesis during autophagy induction.

Very recently, we have demonstrated for the first time that gene expression of human GD3 synthase (hST8Sia I) catalyzing ganglioside GD3 synthesis was upregulated during autophagy induction by curcumin in human lung adenocarcinoma A549 cells [11]. On the contrary, however, we found here that gene expression of human GM3 synthase (hST3Gal V) catalyzing ganglioside GM3 synthesis was downregulated during autophagy induction by curcumin in human colon carcinoma HCT116 cells. The molecular mechanism involved in downregulation of hST3Gal V gene expression in curcumin-stimulated autophagy induction in HCT116 cells was studied.

## 2. Materials and Methods

### 2.1. Materials

Antibodies for Beclin-1, LC3, p62, and CREB were obtained from Cell Signaling Technology (Dancers, Mass, USA); antibody for ATF was purchased from Santa Cruz Biotechnology (Santa Cruz, CA, USA); antibody for glyceraldehydes-3-phosphate dehydrogenase (GAPDH) was purchased from Millipore Corporation (Temecula, CA, USA). Secondary antibodies, horseradish peroxidase-conjugated goat anti-mouse and rabbit Ig G were purchased from Cell Signaling Technology (Dancers, Mass, USA). Curcumin, 4′, 3-methyladenine (3-MA) and compound C (Com C) were purchased from Sigma-Aldrich (St. Louis, MO, USA). Curcumin was dissolved in DMSO at 10 mM for stock concentration and stored at -20°C.

### 2.2. Cell Culture and Viability

Human colon carcinoma HCT116 cells were obtained from American Type Culture Collection (Rockville, MD, USA). Dulbecco's modified Eagle's medium (DMEM; WelGENE Co., Daegu, Korea) containing 10% (v/v) heat-inactivated fetal bovine serum (FBS) and 1% PSA (WelGENE Co., Daegu, Korea) was used as normal growth medium and cells were cultured at 37°C under 5% CO_2_. Cell viability was determined by 3-(4, 5-dimethylthiazol-2-yl)-2,5-diphenyltetrazolium bromide (MTT) assay, as described previously [11, 12].

### 2.3. Immunoblotting Analysis

Immunoblotting analysis was performed as described previously [12]. Cells were lysed in RIPA cell lysis buffer (Pierce, Rockford, IL, USA) containing the protease inhibitor cocktail (Thermo scientific, Waltham, MA, USA). The protein concentration was measured using BCA protein assay kit (Pierce, Rockford, IL, USA). Total proteins were separated by sodium dodecyl sulfate (SDS)-polyacrylamide gel electrophoresis (PAGE), transferred on to a polyvinylidene fluoride (PVDF) membrane (Millipore Corporation, Milford, MA, USA) by electroblotting and then labeled using primary antibodies followed by secondary antibodies. The detection of specific proteins was performed with the Enhanced Chemiluminescence (ECL) Kit (Amersham Biosciences, UK).

### 2.4. Reverse Transcription-Polymerase Chain Reaction (RT-PCR)

Isolation of total RNA from HCT116 cells and RT-PCR experiment including first cDNA synthesis by reverse transcriptase were performed as previously described [11–14]. The primers used in RT-PCR are follows as: hST3Gal V (413 bp), 5′-CCCTGCCATTCTGGGTACGAC-3′(sense) and 5′-CACGATCAATGCCTCCACTGAGATC-3′(antisense); *β*-actin (247 bp), 5′-CAAGAGATGGCCACGGCTGCT-3′ (sense) and 5′-TCCTTCTGCATCCTGTCGGCA-3′ (antisense). PCR products were analyzed by 1.5% agarose gel electrophoresis and visualized with ethidium bromide. The band intensity of the amplified DNA was estimated with a Scion Image Instrument (Scion Corp.; Frederick, MD, USA).

### 2.5. Transfection and Luciferase Reporter Assays

The luciferase reporter plasmids used in the present study, pGL3-83 to pGL3-1600, have been described in previous our studies [12–18]. HCT116 cells were cultured in 24-well plates to about 70% confluency. For transient transfection, the mixtures of serum-free Opti-MEM (Gibco-BRL, Gaithersburg, MD, USA) containing luciferase reporter plasmids (0.5 *μ*g), pRL-TK plasmid (50 ng), and Lipofectamine 2000 (Invitrogen, Carlsbad, CA, USA) were added to each well. After 4 h, the medium was changed to serum-free Opti-MEM and then cultured for 20 h. The next day, the medium was changed to normal growth medium and the cells were treated with 30 *μ*M curcumin for 24 h prior to harvesting. Cells were collected and lysed with passive lysis buffer (Promega, Madison, WI. USA). Firefly luciferase and* Renilla* luciferase activities were quantified with the Dual-luciferase Reporter Assay System (Promega, Madison, WI. USA) using the GlomaxTM 20/20 luminometer (Promega, Madison, WI. USA).

### 2.6. Immunofluorescence

Immunofluorescence staining was conducted as described previously [11]. Briefly, after fixation and blocking of curcumin-treated cells, cells were reacted with the anti-GM3 monoclonal antibody (mouse IgM, Kappa-chain, clone, GMR6; Seigakagu, Tokyo, Japan) followed by FITC-conjugated goat-anti-mouse IgG/M/A mixture (Sigma; St. Louis, MO, USA). The nucleus was stained with DAPI. LSM 700 confocal laser scanning microscope (Carl Zeiss, Oberkochen, Germany) was used to acquire fluorescence images.

### 2.7. Chromatin Immunoprecipitation (ChIP) Assay

ChIP assay was performed with a ChIP assay kit (Millipore, USA) by the same procedures as described previously [12, 14]. DNA samples immunoprecipitated with CREB, ATF and IgG antibodies was used for used as templates for PCR amplification. PCR analysis was carried out using primers flanking the CREB/ATF binding sites in the hST3Gal V promoter: (forward) 5′-GCCCCGGGTGCGTCCCTG-3′ and (reverse) 5′-AGCGCCGCTCTCGCGCC-3′.

## 3. Results

### 3.1. Effect of Curcumin on the Proliferation of HCT116 Cells

**Figure 1:**
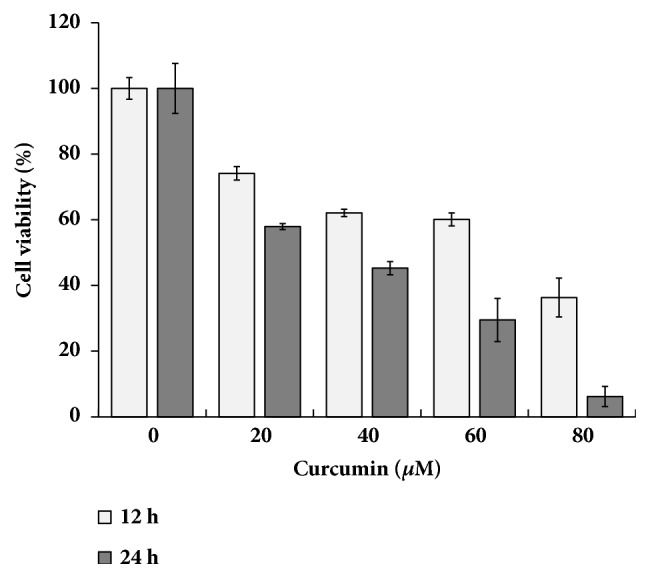
**Effect of curcumin on cell viability of HCT116 cells.** HCT 116 cells were exposed to various concentrations of curcumin (0, 20, 40, 60, 80 *μ*M) for 12 h and 24 h. The cell viability was measured by MTT assay. Data were calculated as percent of vehicle control and expressed as the mean of at least three experiments.

The cytotoxicity of curcumin in HCT116 cells was investigated using MTT assay. HCT116 cells were treated with curcumin in various concentrations for 12 and 24 h. As shown Figure 1, curcumin inhibited dose- and time-dependently HCT116 cell proliferation. Cell viability in cells treated with curcumin for 24 h was significantly decreased compared to those for 12 h.

### 3.2. Effect of Curcumin on Autophagy Induction in HCT116 Cells

**Figure 2:**
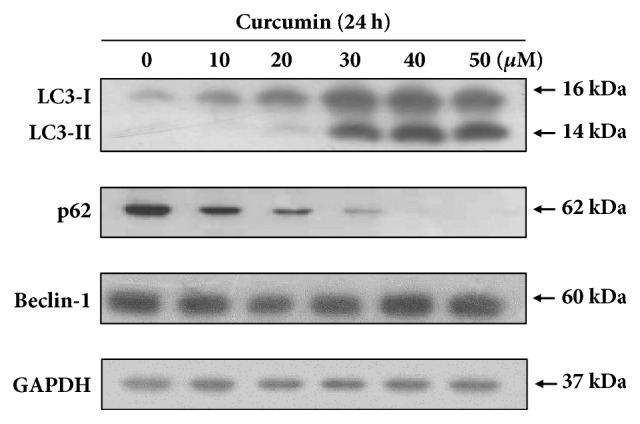
**Effect of curcumin on autophagy induction in HCT116 cells.** HCT116 cells were treated with various concentrations of curcumin (0, 20, 40, 60, 80 *μ*M) for 24 h. Immunoblot analysis was performed using antibodies against LC3, p62, and beclin-1, respectively. GAPDH was used as loading control.

Previous studies have shown that curcumin-induced autophagy in HCT116 cells [19, 20]. To ascertain whether curcumin cause autophagy induction in HCT116 cells, immunoblotting analysis was performed using antibodies to microtubule-associated protein 1 light chain 3 (LC3), Beclin-1 and p62 known as three major autophagy-related markers. As shown in Figure 2, the conversion of LC3-I to LC3-II was remarkably increased at the concentration of 30 *μ*M curcumin, whereas the level of p62 protein was dramatically decreased in a dose-dependent manner. The significant change in Beclin-1 level was not observed. Because the increase of LC3-II level and decrease of p62 level by autophagy induction is well known [21, 22], these results indicate that curcumin induces autophagy in HCT116 cells.

### 3.3. Effect of Curcumin on hST3Gal V Gene Expression in HCT 116 Cells

**Figure 3:**
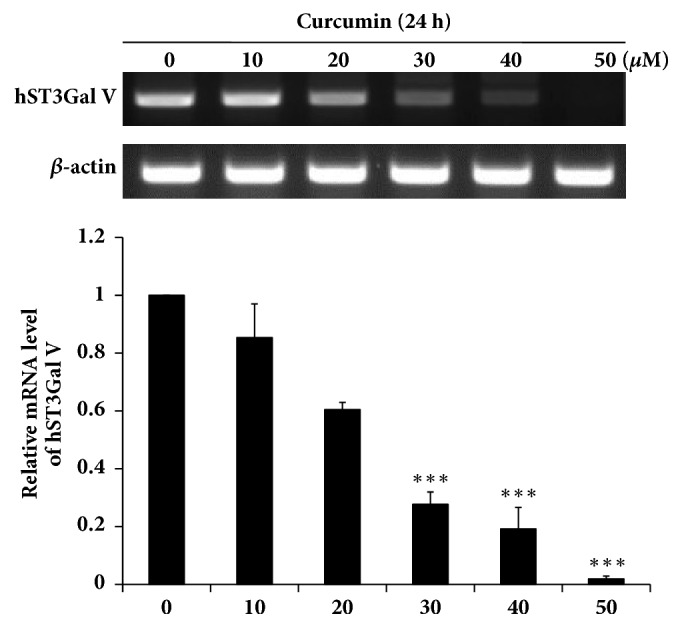
**Effect of curcumin on hST3Gal V mRNA levels in HCT116 cells.** HCT116 cells were treated for 24 h at different concentrations (0, 10, 20, 30, 40, 50 *μ*M) of curcumin, and then hST3Gal V mRNA levels were analyzed by RT-PCR using the extracted total RNAs. The abundance of *β*-actin mRNA was also analyzed as an internal standard. The densitometric intensity of hST3Gal V band was shown as percentages of the control (0 *μ*M) in the panel below. Data represent the relative values ± SEM of three independent experiments and the mean values from each experiment were compared using one-way ANOVA followed by a Newman-Keuls multiple comparison test. *∗∗∗ p* < 0.001* versus* the control.

Our recent report has shown that the level of hST8Sia I mRNA expression was significantly increased in curcumin-treated A549 cells [11]. To investigate whether curcumin can affect hST3Gal V gene expression in HCT116 cells, the hST3Gal V mRNA levels were analyzed by RT-PCR after treatment for 24 h with different concentration of curcumin. As shown in Figure 3, curcumin treatment significantly decreased the levels of hST3Gal V mRNA expression in a dose-dependent manner, and markedly reduced its mRNA levels to 72% of that of untreated control cells at the concentration of 30 *μ*M, indicating that* hST3Gal V gene expression* is transcriptionally downregulated by curcumin in HCT116 cells.

### 3.4. Effect of Curcumin on Ganglioside GM3 Expression in HCT116 Cells

**Figure 4:**
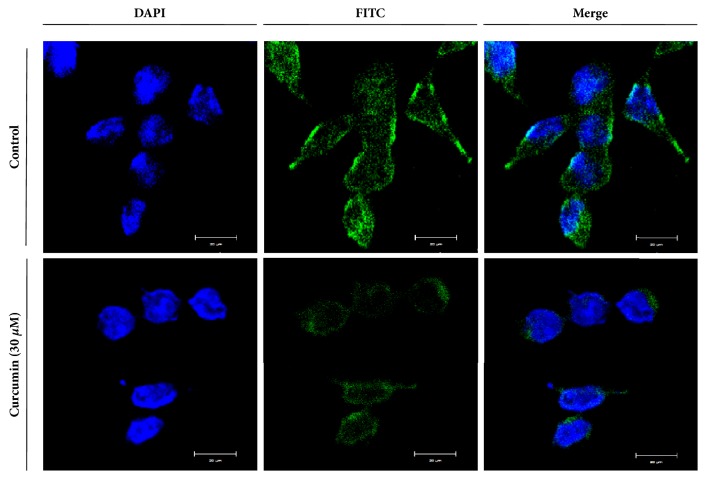
**Immunofluorescence staining of ganglioside GM3 in HCT116 cells treated with curcumin**. Immunostaining using anti-GM3 antibodies (FITC; green) and DAPI staining (blue) were analyzed by confocal laser scanning microscope (400 ×).

To investigate whether or not the decrease of hST3Gal V gene expression by curcumin treatment causes the reduction of ganglioside GM3 level synthesized by hST3Gal V in HCT116 cells, the level of cellular expression of ganglioside GM3 was determined by immunofluorescence confocal microscopy using anti-GM3 mAb and FITC-conjugated anti-mouse IgG/M/A mixture as secondary. As shown in Figure 4, ganglioside GM3 expression was significantly decreased in HCT116 cells treated with 30 *μ*M curcumin for 24 h, compared with curcumin-untreated control HCT116 cells.

### 3.5. Analysis of hST3Gal V Promoter Region Responsive to Curcumin in HCT116 Cells

**Figure 5:**
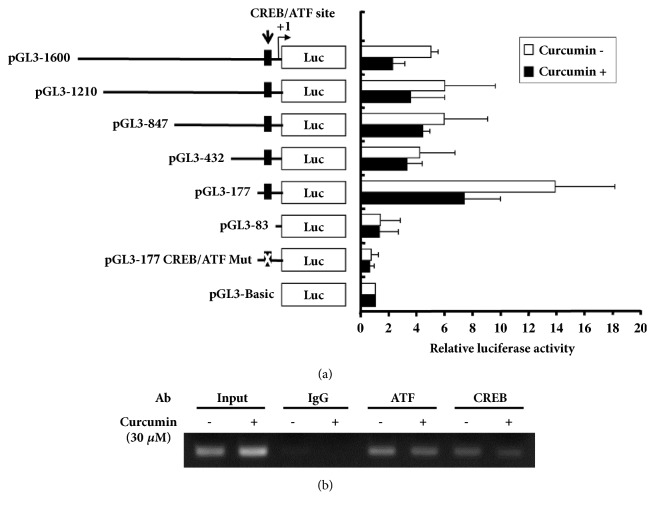
**Analysis of the hST3Gal V promoter activity and ChIP assay in HCT116 cells treated with curcumin**. (a) Schematic representation of DNA constructions containing different 5′-deletion of the promoter region of the hST3Gal V linked to the luciferase reporter gene is presented on the left. Relative luciferase activities obtained in control (open bar) or curcumin-treated cells (solid bar) are shown on the right and were normalized to the* Renilla* luciferase activity derived from pRL-TK. Data are presented as the means ± SD of three independent experiments with triplicate measurements. (b) ChIP assay performed in curcumin-treated HCT116 cells, or nontreated cells with input control (without antibody) and nonspecific immunoglobulin (IgG), CREB, and ATF antibodies. The precipitated chromatin was amplified by PCR with primers specific for the CREB/ATF consensus binding site on hST3Gal V promoter.

To assess whether curcumin regulates hST3Gal V gene expression through transcriptional regulation in HCT cells, transient transfection experiments of luciferase reporter genes with a series of 5′-truncated constructs of the hST3Gal V promoter were performed in HCT116 cells (Figure 5(a)). As shown in Figure 5(a), cells transfected with the pGL3-177 construct showed the strongest activity for the hST3Gal V promoter, which was about 12-fold relative to the pGL3-Basic control vector, and curcumin treatment caused a 48% decrease in promoter activity. However, promoter activities obtained with four 5′-truncated constructs (pGL3-1600 to pGL3-432) were significantly lower (about 7- to 9-fold) than that with pGL3-177 construct, suggesting that the -1600 to -177 region contains potential negative regulatory elements for transcriptional activity, as shown in previous reports [12–18]. Furthermore, promoter activity obtained with pGL3-83 construct showed a remarkable reduction, which was the similar level of the pGL3-Basic control vector. These results clearly suggest that the region -177/-83 plays a crucial role for the expression of hST3Gal V gene and functions as the curcumin-responsive promoter in HCT116 cells.

Our previous studies have demonstrated that that a putative CREB/ATF consensus binding site (5′-TGACGTCA-3′) located at position -143 in the region -177/-83 is essential for the expression of hST3Gal V gene in various type of cells [13, 14, 16–18].

Thus, we next investigated whether CREB/ATF was involved in the expression of the hST3Gal V gene induced by curcumin. The pGL3-177 CREB/ATF Mut construct with a mutation at CREB/ATF site was transfected into HCT116 cells and promoter assay was carried out. As shown in Figure 5(a), the promoter activity obtained with pGL3-177 CREB/ATF Mut was lower than that with the pGL3-Basic vector regardless of curcumin treatment, indicating that destruction of the CREB/ATF site affected the promoter activity significantly. This result suggests that this CREB/ATF site is essential for the expression of hST3Gal V gene in HCT116 cells. To confirm that CREB and ATF interact with the hST3Gal V promoter in vivo, we performed ChIP experiments with CREB and ATF antibodies and nuclear extracts from HCT116 cells treated with curcumin. After formaldehyde cross-linking, sonication, and precipitation of the chromatin with CREB, ATF, or IgG antibodies, the precipitated DNA was subjected to PCR amplification using primers flanking the CREB/ATF binding site on the hST3Gal V promoter. As shown in Figure 5(b), the amplified products were observed in CREB and ATF immunoprecipitations, whereas no amplified product was detected in IgG immunoprecipitation, indicating that CREB/ATF expressed in HCT116 cells had the ability to bind specifically to the CRE/ATF site of hST3Gal V promoter. On the other hand, the amplified signal in CREB and ATF immunoprecipitations in curcumin-treated cells was decreased to about 45% and 58% of that of untreated cells, respectively. This result indicated that curcumin suppressed the transcription of hST3Gal V gene, at least in part, by inhibiting CREB/ATF-mediated transcriptional activity.

### 3.6. Transcriptional Activation of hST3Gal V Gene via AMPK Pathway in HCT116 Cells

**Figure 6:**
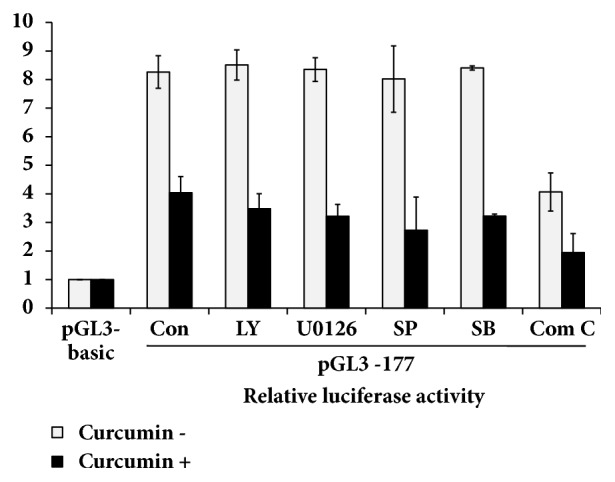
**Effect of curcumin on signaling pathway responsible for transcriptional activation of hST3Gal V in HCT116 cells.** The pGL3-177 (positive control), pGL3-basic (negative control), and pRL-TK (internal control) were cotransfected into HCT116 cells. Transfected cells were incubated in the presence (solid bar) and absence (open bar) of 30 *μ*M curcumin with LY294002 (10 *μ*M), U0126 (10 *μ*M), SP600125 (5 *μ*M), GŐ6983 (100 nM), and compound C (10 *μ*M) inhibitors for 24 h. Relative luciferase activity was normalized with the* Renilla* luciferase activity derived from pRL-TK. Data represent mean ± SEM for three independent experiments with triplicate measurements.

We next investigated the signal transduction pathway responsible for transcriptional activation of hST3Gal V gene expression in HCT116 cells. As shown in Figure 6, promoter activity of pGL3-177 as a control was significantly suppressed in curcumin-treated HCT116 cells, compared with untreated HCT116 cells. This pattern was not significantly affected by LY294002 (PI3K/AKT inhibitor), U0126 (MEK/ERK inhibitor), SP600125 (JNK inhibitor), and SB203580 (p38 MAPK inhibitor), whereas compound C (AMPK inhibitor) treatment resulted in a 50% decrease in promoter activity. These results indicate that transcriptional activation of hST3Gal V gene in HCT116 cells is mediated by AMPK signaling pathway.

### 3.7. Effect of 3-MA on hST3Gal V Gene Expression in HCT116 Cells

**Figure 7:**
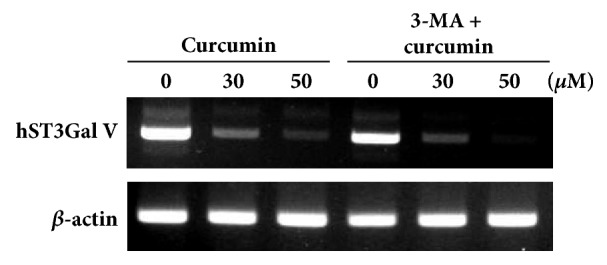
**Effect of 3-MA on curcumin-induced hST3Gal V gene expression in HCT116 cells. **HCT116 cells were preincubated with or without 3-MA (10 mM) for 3 h and then incubated with curcumin (0, 30, 50 *μ*M) for 24 h. The hST3Gal V mRNA levels were analyzed by RT-PCR using the extracted total RNAs. The abundance of *β*-actin mRNA was also analyzed as an internal standard.

To explore that autophagy induction by curcumin has an effect on curcumin-induced suppression of hST3Gal V gene expression in HCT116 cells, cells were treated with 3-methyladenine (3-MA), a specific autophagy inhibitor, for 3 h before curcumin treatment and the hST3Gal V mRNA levels were analyzed by RT-PCR. As shown in Figure 7, 3-MA did not inhibit the curcumin-induced suppression of hST3Gal V gene expression, indicating that curcumin-induced downregulation of hST3Gal V gene expression is independent of autophagy induction by curcumin in HCT116 cells.

## 4. Discussion

In the present study, we first demonstrated that curcumin downregulates human GM3 synthase (hST3Gal V) gene expression with autophagy induction in HCT116 human colon cancer cells. The present finding is opposite to our recent result that curcumin upregulates human GD3 synthase (hST8Sia I) gene expression with autophagy induction in human lung adenocarcinoma A549 cells [11]. Given that hST3Gal V and hST8Sia I are key enzymes catalyzing the first step in the synthesis of a-, b-, and c- series gangliosides [2–4], it is striking that curcumin shows the opposite effects on expression of these genes with autophagy induction. In addition, our result obtained in this study is also inconsistent with previous our results showing that hST3Gal V gene expression was upregulated by valproic acid, a simple branched-chain fatty acid, in SK-N-BE(2)-C human neuroblastoma cells [13] and ARPE-19 human retinal pigment epithelial cells [14].

Our present data showed that a 94-bp fragment located between positions -177 and -83 in the hST3Gal V promoter, including the putative CREB/ATF consensus binding site, contains regulatory elements that mediate, at least partially, transcriptional repression by curcumin in HCT116 cells. In previous studies, we have demonstrated that upregulation of hST3Gal V gene by valproic acid, or during monocytic differentiation of HL-60 cells by 12-O-tetradecanoylphorbol-13-acetate (TPA) was transcriptionally mediated by the consensus CREB/ATF binding site at position -143 to -136 of hST3Gal V gene promoter [13, 14, 16–18]. Similarly, the current result obtained by site-directed mutagenesis and in vivo ChIP assay demonstrated that downregulation of hST3Gal V gene expression by curcumin in HCT116 cells was also mediated by this CREB/ATF binding site.

Furthermore, we have previously demonstrated that transcriptional activation of the hST3Gal V gene by TPA in HL60 cells occurs through PKC/ ERKs signal transduction pathway [17], whereas JNKs signaling pathway is associated with transcriptional activation of the hST3Gal V gene by valproic acid in ARPE-19 cells [14]. However, our current data indicate that none of these signaling pathways contribute to the hST3Gal V gene expression by curcumin in HCT116 cells, whereas AMPK signaling pathway is related to transcriptional expression of the hST3Gal V gene in curcumin-stimulated HCT116 cells. These findings are in accordance with our previous results showing the signal transduction pathway that leads to transcriptional activation of hST8Sia I gene expression in curcumin-stimulated A549 cells [11]. In addition, curcumin was recently reported to induce AMPK activation in human colon cancer SW 480 cells [23] and human ovarian cancer CaOV3 cells [24].

It was previously reported that gangliosides were associated with autophagy induction [10, 25]. Therefore, it is important to elucidate whether autophagy induction by curcumin in HCT116 cells is related to the downregulation of hST3Gal V gene expression. Our present results provide direct evidence that the repression of hST3Gal V gene expression is due to curcumin, but not autophagy induction by curcumin as demonstrated by the use of 3-MA, a specific autophagy inhibitor.

## 5. Conclusion

We have first shown that curcumin downregulates human GM3 synthase (hST3Gal V) gene expression with autophagy induction in HCT116 human colon cancer cells. The CREB/ATF binding site at -143 was shown to be crucial for transcriptional regulation of hST3Gal V gene in response to curcumin in HCT116 cells. Our results suggest that transcriptional activation of hST3Gal V gene in HCT116 cells is mediated by AMPK signaling pathway and downregulation of hST3Gal V gene expression is not associated with autophagy induction in curcumin-treated HCT116 cells.

## Data Availability

The data used to support the findings of this study are available from the corresponding author upon request.
